# A sudden and unprecedented increase in low dose naltrexone (LDN) prescribing in Norway. Patient and prescriber characteristics, and dispense patterns. A drug utilization cohort study

**DOI:** 10.1002/pds.4110

**Published:** 2016-09-26

**Authors:** Guttorm Raknes, Lars Småbrekke

**Affiliations:** ^1^ Regional Medicines and Information and Pharmacovigilance Centre (RELIS)University Hospital of North NorwayTromsøNorway; ^2^ National Centre for Emergency Primary Health CareUni Research HealthBergenNorway; ^3^ Department of Pharmacy, Faculty of Health SciencesUiT—The Arctic University of NorwayTromsøNorway

**Keywords:** low dose naltrexone, pharmacoepidemiology, drug utilization study, prescription database study, off‐label prescribing, pharmacoepidemiology

## Abstract

**Purpose:**

Following a TV documentary in 2013, there was a tremendous increase in low dose naltrexone (LDN) use in a wide range of unapproved indications in Norway. We aim to describe the extent of this sudden and unprecedented increase in LDN prescribing, to characterize patients and LDN prescribers, and to estimate LDN dose sizes.

**Methods:**

LDN prescriptions recorded in the Norwegian Prescription Database (NorPD) in 2013 and 2014, and sales data not recorded in NorPD from the only Norwegian LDN manufacturer were included in the study.

**Results:**

According to NorPD, 15 297 patients (0.3% of population) collected at least one LDN prescription. The actual number of users was higher as at least 23% of total sales were not recorded in NorPD. After an initial wave, there was a steady stream of new and persistent users throughout the study period. Median patient age was 52 years, and 74% of patients were female. Median daily dose was 3.7 mg. Twenty percent of all doctors and 71% of general medicine practitioners registered in Norway in 2014 prescribed LDN at least once.

**Conclusions:**

The TV documentary on LDN in Norway was followed by a large increase in LDN prescribing, and the proportion of LDN users went from an insignificant number to 0.3% of the population. There was a high willingness to use and prescribe off label despite limited evidence. Observed median LDN dose, and age and gender distribution were as expected in typical LDN using patients. © 2016 The Authors. *Pharmacoepidemiology and Drug Safety* Published by John Wiley & Sons Ltd.

## Introduction

Naltrexone is an opioid antagonist originally used to treat opioid and alcohol addiction.^1^, ^2^ Over the past two decades, low dose naltrexone (LDN—typically <5 mg/day) has gained popularity as an off‐label treatment of several autoimmune diseases.^3^ Some studies including a limited number of patients and with short duration of follow‐up are inconclusive on efficacy in multiple sclerosis (MS), Crohn's disease, chronic pain, and fibromyalgia.^4^, ^5^, ^6^, ^7^ In February 2016, LDN was rated as the most effective among 35 MS treatments at CureTogether, an online social network for patients.^8^ No studies have described patients receiving long time off‐label LDN therapy, discontinuation rates, or proportion of persistent users. There are no guidelines on LDN dosing, but some authors suggest daily doses of 3–4.5 mg.^9^ To our knowledge, the actual doses taken by LDN using patients have not been characterized in pharmacoepidemiological studies. LDN is relatively unknown among doctors and should be considered as alternative or complementary therapy because high quality clinical and epidemiological studies are lacking.

Several possible mechanisms of action have been suggested, mostly in which endogenous opioids play a role.^9^ Naltrexone has been used for decades, and the safety profile when used for approved indications is well known.

Naltrexone is a low‐cost generic drug with limited commercial potential. If proven efficacious for one or more of the mentioned patient groups, it will be a cheap supplement or competitor to several standard treatments.

On 27 February 2013, the biggest commercial TV‐channel station in Norway (TV2) aired a documentary on the alleged effects of LDN. Patients with severe MS explained how the use of LDN had almost normalized their function.^10^ Following the documentary, there was a large increase in the awareness and consumption of LDN in Norway among patients with a wide range of diagnoses. In social media, Norwegian LDN groups gained thousands of new members within few weeks. A preliminary search in the Norwegian Prescription Database (NorPD) revealed that the number of naltrexone users increased from 14 in 2012 to more than 11 000 in 2013.^11^ The sudden increase in the number of users sheds light on mass media impact on patient and prescriber behavior when it comes to alternative or complementary medical treatments.

The main objectives of this study were to determine the extent of this sudden and unprecedented tsunami like increase in LDN prescribing by characterizing the LDN prescription patterns, and to calculate the mean daily dose among persistent users. The secondary objective was to describe the prescriber demographics and specialties.

## Methods

### Study design and data sources

This drug utilization cohort study used data from the NorPD on dispensed prescriptions of LDN covering the entire Norwegian population. In addition, we obtained sales statistics for 2013 and 2014 from the only Norwegian manufacturer of naltrexone 3‐mg tablets to account for unrecorded prescribing of LDN.

NorPD contains individual data on all prescriptions dispensed since 2004 to the entire Norwegian population living outside institutions (hospitals and nursing homes). Details on NorPD are published elsewhere.^12^ In short, NorPD contains a unique pseudonym for the personal identifier and demographic data on patient and prescriber, the specialty of the prescriber, the Anatomical Therapeutic Chemical (ATC)‐code and the amount of drug prescribed, date of dispensing, and location of the dispensing pharmacy. It is possible to follow prescriptions to individual patients, and both reimbursed and non‐reimbursed prescriptions are recorded. Only prescriptions on products that have a product identifying number are recorded, and preparations compounded by pharmacies according to a physician's prescription are not included. The database contains no information on the indication for therapy, but has disease codes (ICD‐10 or ICPC‐2) for reimbursed medications. The database is operated by the Norwegian Institute of Public Health.^13^ We used the following variables in this study: person identifier for patient and prescriber, patient and prescriber age and sex, prescriber specialty, ATC‐code, product number, date of dispensing, and dispensed volume in defined daily doses (DDD). Prescriptions are recorded in NorPD only when they are actually dispensed. For a fee, NorPD provided a data file according to their data access procedures.

All patients included in NorPD with a prescription on LDN (ATC‐code N07B B04) in 2013 or 2014 were included in the cohort, and all recorded LDN prescriptions from 1 January 2013 through 31 December 2014 were included. Only one LDN product (Naltrekson Kragerø 3‐mg tab, Kragerø tablettproduksjon AS) was recorded. This product was assigned a product identifying number (361181) on 15 May 2013. Incident use was identified by the first patient specific appearance of this product number in NorPD.

### Outcomes

The main outcome was the number of LDN prescriptions in NorPD. In order to estimate LDN daily doses and time between dispenses, we followed patients that repeatedly collected LDN prescriptions. Average daily dose was calculated based on number of days between repeated dispenses and amount dispensed. We defined patients retrieving five or more naltrexone prescriptions as persistent users.

We calculated median age and gender distribution for all prescriptions. For patients collecting more than one prescription, we calculated time since last dispense. We identified the total number of prescribers and corresponding specialties, both in absolute numbers and as proportions of all doctors.

Study size was determined by the number of dispensed LDN prescriptions in 2013 and 2014.

The specialist doctor classification is in accordance with the official EU harmonized Norwegian specialty structure.^14^

### Statistical methods

Data were processed in Microsoft Excel 2010, SPSS 23, and StataMP 14. No statistical tests were used, because the total population was covered. Percentiles (10 and 90 and median) for age and average daily dose were calculated.

### Ethics

The project protocol was submitted to the Regional Committee for Medical and Health Research Ethics of Northern Norway. The committee concluded that disclosure was not mandatory, because the data were pseudonymized. The local privacy ombudsman for research at the University Hospital of Northern Norway approved the project. For Norwegian central health registries like NorPD, consent from individual patients is by law not required.

## Results

In 2013 through 2014, 15 297 patients (0.3% of total population in Norway) collected 48 006 boxes of 100 tablets of LDN from 45 216 prescriptions at Norwegian pharmacies according to NorPD. In addition, the manufacturer sold 14 506 boxes (23.2% of total sales) directly to pharmacies and wholesalers before the product identification number was assigned on 15 May 2013.^15^ This amount was not captured by NorPD. Prior to 2013, Kragerø tablettproduksjon had an average monthly sale of approximately 100 boxes of LDN to wholesalers or local pharmacies, also not captured by the NorPD.^15^

In the NorPD database, there were 18 500 dispenses to 11 247 unique patients in 2013, of which 6320 (56%) also retrieved at least one LDN prescription in 2014. In 2014, there were 26 716 dispenses to 10 370 unique patients. Total NorPD observation time (from first LDN collected to 31 December 2014) was 247 478 patient months, of which 140 158 patient months for individuals that collected LDN more than once.

**Figure 1:**
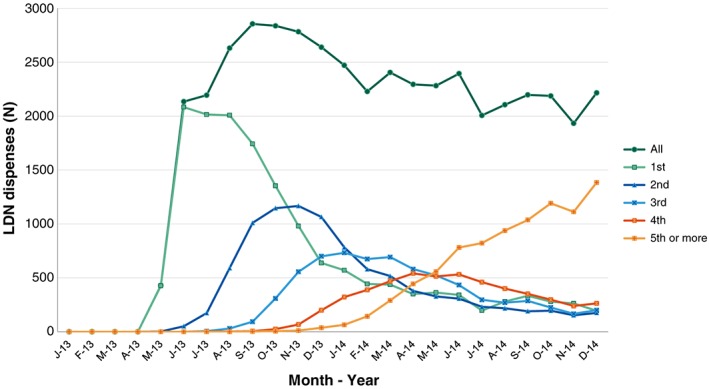
Dispensed low dose naltrexone (LDN) prescriptions recorded in Norwegian Prescription Database (NorPD) by month. Total and repeated prescriptions. A documentary on LDN was aired 27 February 2013, and a LDN product was first included in NorPD from 15 May 2013 [Color figure can be viewed at wileyonlinelibrary.com]

Figure 1 shows the number of LDN dispenses in NorPD by month. After the TV documentary, the number of monthly dispenses reached a maximum of 2855 in September 2013. The number of dispensed LDN prescriptions then stabilized between 2000 and 2500 per month. First time dispenses (incident use) reached a maximum of 2083 in June 2013, but stabilized around 2–300 per month throughout 2014. In addition, the number of patients with five or more naltrexone dispenses or more has increased steadily since December 2013.

Mean and median patient age was 52 years, range 4 to 102 years, and 74% of patients were female. Eighty children under 16 years were dispensed 161 LDN prescriptions. Altogether, 9256 patients (60.5% of total) collected two or more LDN prescriptions in the study period. For repeated LDN prescriptions, median time between two dispenses were 83 days, mean 92 days. There was a decreasing proportion of females, increasing age, and decreasing time since last dispense with increasing number of dispenses (Table 1).

**Table 1:** Frequencies of first (incident) and repeated and low dose naltrexone (LDN) prescriptions, with age and gender distribution. Time since last dispense for repeated LDN prescriptions

Collected LDN prescription #	*N*	% females	Patient age				Time since last LDN dispense (days)			
			Mean	Median	10 percentile	90 percentile	Mean	Median	10 percentile	90 percentile
1	15 297	73.8	50.4	51	32	68	—	—	—	—
2	9256	73.9	51.3	51	34	68	103.8	92	53	167
3	6770	74.0	52.1	52	35	69	99.2	89	52	161
4	5074	74.6	52.6	53	36	69	93.7	86	49	147
5	3616	74.6	52.9	53	37	69	85.5	79	44	130
6	2291	74.6	53.3	53	37	69	78.6	73	44	120
7	1348	73.4	53.3	53	38	69	69.3	68	35	104
8	719	72.5	53.9	53	39	70	61.9	63	24	93
9	308	70.1	55.2	54	40	71	52.2	54	15	79
10 or more	537	65.9	62.0	55	42	71	23.5	43	13	73
All	45 216	73.9	51.7	52	34	69	92.4	83*	44*	147*

* For dispense # > 1.

**Figure 2:**
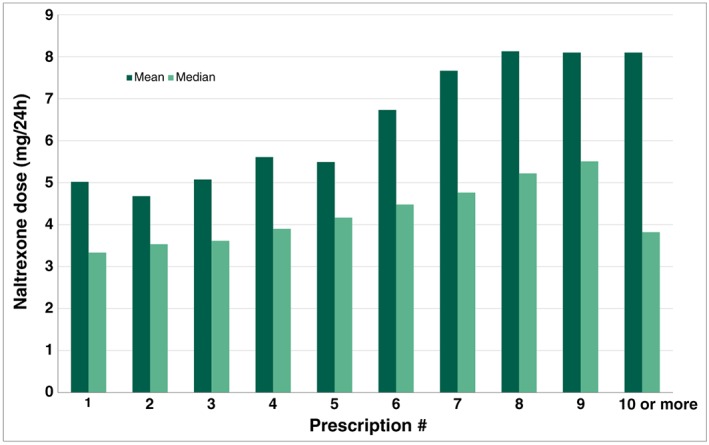
Median and mean naltrexone doses for all patients collecting more than LDN prescription [Color figure can be viewed at wileyonlinelibrary.com]

Median estimated daily dose based on dispensed amount and time between dispenses was 3.7 mg, mean 5.3 mg. Increasing dispense number was associated with increased dose, as shown in Figure 2.

LDN dispenses were from 4695 different prescribers. This accounts for 19.7% of all working doctors registered in Norway in 2014.^16^ Fifteen per cent of the prescribers accounted for 50% of the dispensed prescriptions. Median number of LDN prescriptions per prescriber was 5 and 90% prescribed 22 LDN prescriptions or less. There were 939 (19.1%) one‐time prescribers. Fourteen doctors had more than 100 LDN prescriptions. One doctor prescribed LDN 569 times, accounting for more than 1% of all LDN prescriptions in Norway in 2013 and 2014. See Table 2 for prescriber characteristics. Specialists constituted 57% of all prescribers, and 82.6% of LDN prescribing specialists were general practitioners.

**Table 2:** Age and gender of Norwegian LDN prescribers by specialist status.

	All prescribers			Certified specialists			Prescribers without specialty		
	*N*	%	Mean age	*N*	%	Mean age	*N*	%	Mean age
All prescribers	4695	100	46.0	2668	100	52	2027	100	38.1
Male	2823	60.1	48.4	1771	66.4	53.5	1052	51.9	39.9
Female	1868	39.8	42.5	893	33.5	49.3	975	48.1	36.2
Age and gender unknown	4	0.1	NA	4	0.1	NA	0	0	—

The mean age of the LDN prescriber was 46 years, 1.5 years older than the average working doctor in Norway. Female doctors accounted for 39.8% of LDN prescribers, but 33.5% of LDN prescriptions, both lower than the proportion of working female doctors in Norway (44.5%). Male prescribers were older than the female, both among specialists and non‐specialists. Specialties representing the most frequent prescribers are presented in Table 3. More than 71% of general medicine specialists wrote at least one prescription. Among specialists in public health, gastroenterology, rheumatology, and among specialists in physical medicine and rehabilitation, more than one in 10 were LDN prescribers.

**Table 3:** The 15 most frequent medical specialties among LDN prescribers. Frequency and proportion of LDN prescribers within each specialty in Norway (%)

Specialty	LDN prescribers	
	*N*	%
General practice	2290	71.4
Public health	147	26.9
Internal medicine	116	6.0
Psychiatry	46	2.9
Gastroenterology	38	14.7
Neurology	37	8.3
Pediatrics	33	4.7
Anesthesiology	32	3.2
General surgery	29	2.3
Physical medicine and rehabilitation	23	10.8
Rheumatology	23	10.8
Gynecology and obstetrics	21	2.7
Occupational health	18	6.7
Orthopedic surgery	16	2.5

## Discussion

### Key results

In this study, we have quantified the extent and identified prescription patterns of the unprecedented increase in LDN prescribing following a Norwegian TV documentary in 2013. According to NorPD, 15 297 patients collected a total number of 45 216 LDN prescriptions in 2013 and 2014, compared to almost zero in the preceding years. In addition, the manufacturer sold 14 506 boxes of LDN that were not captured by NorPD. The maximum monthly number of NorPD recorded LDN prescriptions reached 2855 after the TV documentary, and the number of LDN prescriptions to new users in NorPD stabilized at 2–300 per month. Sixty per cent of the patients collected two or more LDN prescriptions, and during the 2‐year study period, the number of patients that collected five or more prescriptions increased. Of LDN prescriptions in NorPD, 74% were dispensed to females, and median age of patients at the time of the first dispense was 52 years. Median time between repeated dispenses was 83 days. We estimated a median daily naltrexone dose of 3.7 mg for patients that retrieved more than one prescription. One in five doctors registered in Norway prescribed LDN in 2013 or 2014.

### Interpretation

The TV documentary was followed by an extreme increase of dispensed LDN prescriptions, and our study indicates that LDN has become more than an ephemeral phenomenon in Norway. LDN was regularly used as off‐label treatment by many patients, and there was a steady recruitment of new users throughout 2014. According to the manufacturer, the LDN annual sale is relatively stable. In 2015, 22 511 boxes were sold to wholesalers.^15^ The number of LDN users in Norway increased from a handful individuals to approximately 3000 per million in 2013 and 2014. One milligram of naltrexone (low dose formulation) was priced to approximately NOK 1.33 (USD 0.15), which means that the Norwegian LDN market in 2014 was equivalent to approximately USD 260 000 per million capita. Autoimmune diseases are more common in women, and the high proportion of female LDN users is therefore as expected. In addition, there is some evidence suggesting that LDN might be efficacious against fibromyalgia and similar conditions.^6^, ^7^ LDN probably gained attention and popularity in these female‐dominated patient groups in Norway after the TV documentary. The observed median age is as expected for these patient groups. The overall estimated median naltrexone dose of 3.7 mg is representative for typical LDN doses, which according to the largest Norwegian LDN user forum (www.ldn.no), range from 0.75 mg to 6 mg per day.^17^ Mean doses are higher than median doses, reflecting a small number of high dose naltrexone users. We observed increased LDN doses with increasing number of dispenses. One possible explanation is that some patients tried increasing doses to get the desired effect.

Most doctors only wrote one or two prescriptions, leaving most of the LDN prescribing to a limited number of doctors. Considering the massive attention LDN has received among MS patients, the proportion of prescribing neurologists is lower than we expected.

We have not identified other pharmacoepidemiological studies on LDN, and the few clinical studies are mostly uncontrolled, or studies with few participants. To our knowledge, this Norwegian sudden surge in LDN use is unparalleled, and Norway probably had the world's highest LDN prescription rate in 2013 and 2014.

The high proportion of persistent LDN users is of considerable interest and warrants further studies. This group should be characterized on indication for use, possible comorbidities, and concurrent medication use.

A majority of Norwegian GPs prescribed LDN in the study. This indicates a high willingness to prescribe off‐label with limited evidence of safety and efficacy. This impression is reinforced by the fact that LDN was prescribed to children as young as 4 years.

### Limitations

The completeness of NorPD is normally considered as a major strength. Unfortunately, the LDN product was not assigned a national product identity number until 15 May 2013, and the volume sold before this date cannot easily be linked to patients or prescribers. Consequently, the number of users and prescribers is probably higher than the numbers reflected from the NorPD, and we are unable to identify incident users with certainty. We observed a maximum of LDN dispenses in September 2013, but the use (including unrecorded LDN) probably peaked earlier. In the first weeks following the TV documentary, the situation was extraordinary. Shortly after the documentary, the Norwegian manufacturer of LDN tablets was unable to meet the demand, and possibly some patients diluted 50‐mg naltrexone tablets for LDN use according to instructions available on the Internet.^18^, ^19^ However, prescriptions in NorPD for such use were indistinguishable from naltrexone to approved indications and were not included in our material. Private import from internet pharmacies or pharmacies abroad may have occurred, but the extent of this is unknown. From the second half of 2013 and for 2014, the producer was able to supply the market, and LDN use from sources not captured by NorPD was probably negligible. In most cases, the dispensed amount of LDN was one box of 100 tablets.

The number of persistent users is estimated from NorPD data and is probably underestimated in our study. We have defined persistent use as collection of five or more LDN prescriptions. With the observed median daily dose, five collected boxes of LDN would be enough for approximately half the days in the study period. However, there were patients in our material who collected LDN with very long time intervals. Some probably used as little as a quarter of a tablet per day, which means that one box lasted for 400 days. These were not identified as persistent users despite probable continuous use for more than a year. Towards the end of the study period, there were also incident users where observation time was too short to identify persistence.

We emphasize that we have neither examined indications, nor the efficacy or safety of LDN use. Many patients became persistent users of LDN, and this may be interpreted as a perceived acceptable effect and safety profile among these patients. This cannot be considered as a proof of LDN treatment efficacy for any condition. Still, the persistent use among thousands of LDN users and the widespread willingness to prescribe LDN are clear signals that robust clinical studies on untraditional use of naltrexone are needed.

## Conclusion

Our study demonstrates how a single TV documentary combined with “viral” spread on social media most likely had a massive impact on patient LDN demand and prescriber behavior. Similar LDN “tsunamis” may hit other shores at any time.

## Conflict of Interest

Both authors have completed the Unified Competing Interest form at http://www.icmje.org/coi_disclosure.pdf (available on request from the corresponding author) and declare: No support from any organization for the submitted work.

None of the methods, results, or contents of this manuscript has been published, posted, or presented in any form.

**Key Points**

- Low dose naltrexone (LDN) is used as off‐label treatment of several autoimmune and other diseases.
- A TV documentary in 2013 probably led to a huge increase in the awareness and consumption of LDN in Norway.
- More than 15 000 new LDN users in Norway were recorded after the TV documentary, most of them collected more than one prescription.
- Among persistent LDN users, median daily dose was 3.7 mg.
- LDN was prescribed at least once by 71% of Norwegian general practitioners, indicating a great willingness to prescribe off label with limited evidence of efficacy.

## Funding

The study was funded by RELIS Nord‐Norge and UiT—The Arctic University of Norway.

## Author contributions

GR contributed to the conception of the study, designed the study, performed statistical analyses, and drafted the manuscript. LS contributed to the design of the study and to statistical analyses. Both authors participated in revising the manuscript and approving the final version.
